# Editorial: Lipid Signaling in Plant Development and Responses to Environmental Stresses

**DOI:** 10.3389/fpls.2016.00324

**Published:** 2016-03-17

**Authors:** Eric Ruelland, Olga Valentova

**Affiliations:** ^1^Université Paris-Est, Institut d'Ecologie et des Sciences de l'Environnement de ParisCréteil, France; ^2^Centre National de la Recherche Scientifique, Unité Mixte de Recherche 7618, Institut d'Ecologie et des Sciences de l'Environnement de ParisCréteil, France; ^3^Department of Biochemistry and Microbiology, University of Chemistry and Technology, PraguePrague, Czech Republic

**Keywords:** lipid signaling pathways, phospholipases, phosphatidic acid, diacylglycerol pyrophosphate, lipid-kinases, phosphoinositides, inositol phosphates

In response to environmental stresses, or during development, plant cells will produce lipids that will act as intracellular mediators (Janda et al., 2013; Ruelland et al., 2015). Glycerophospholipid and/or sphingolipid second messengers resulting from the action of lipid metabolizing enzymes (e.g., lipid-kinases or lipases) are commonly found within cells. The articles published within this Research Topics clearly illustrate the trends of this research field that we can sum up as follows.

## The use of mutant approach to further understand the roles of lipid signaling

A major contribution to understanding the role of phosphatidylinositol 3-kinase (PI3K), which catalyses the formation of phosphatidylinositol 3-phosphate (PI3P) from phosphatidylinositol, has been provided in this special issue. The PI3K inhibitor, LY294002, affects PI3P levels *in vivo* and triggers a decrease in proline accumulation in response to salt treatment of *Arabidopsis thaliana* seedlings. This is correlated with a higher transcript and protein levels of *Proline dehydrogenase 1* (*ProDH1*), a key-enzyme in proline catabolism. Interestingly the *ProDH1* expression is induced in a *pi3k*-hemizygous mutant, which clearly illustrates the role of PI3P in response to abiotic stresses (Leprince et al.). PI3P can be phosphorylated into phosphatidylinositol (3,5)-bisphosphate [PtdIns(3,5)P_2_] by Fab1 phosphatidylinositol-3-phosphate 5-kinases. A reverse genetic approach by Malhó et al. has shown that these enzymes, localized to the endomembrane compartment, are involved in the regulation of plasma membrane recycling events and thus in the maintenance of polarity of pollen tube growth. However, a reverse genetic approach in plants can be tricky. In Arabidopsis, many enzymes of the lipid signaling machinery are encoded by multigenic families. For example, phospholipase D (PLD) has 12 gene members in Arabidopsis. In their study on plants response to pathogens, Johansson et al. elegantly combine a pharmacological approach with a reverse genetic one in order to cope with the PLD family redundancy. They clearly show that the decrease of PLD-dependent phosphatidic acid (PA) production by *n*-butanol strongly inhibits the hypersensitive response following *Pseudomonas syringae* effector recognition. Yet, the screening of different *pld* mutants (single or multiple mutants) in response to *P. syringae* AvrRpm1 recognition did not allow the identification of isoforms associated with the induction of HR following recognition. The authors reasoned that the PLD activity in response to AvrRpm1 recognition is therefore likely caused by the activation of several PLD isoforms and that the individual contributions might be so small that the single knock-outs show no phenotype. Yet, if the overall activity of PLD is lowered by addition of *n*-butanol, it might be possible to detect the effect of loss of single PLD isoforms. Doing so, i.e., screening again their mutants in response to AvrRpm1 recognition but in presence of *n*-butanol, they could identify several mutants for which the hypersensitive response was lower than that of WT plants (in presence of *n*-butanol). These mutants are impaired in isoforms that in WT plants participate in the bulk PLD activity downstream of AvrRpm1 recognition.

Another limitation of reverse genetic occurs in gene families with only a few members, for which a reverse genetic approach can result in the lack of production of viable mutants. This is illustrated by the *impl1* mutant that is mutated in the *myo*-inositol monophosphatase (IMP) enzyme that hydrolyses D-*myo*-inositol 1-phosphate, a breakdown product of D-inositol (1,4,5) trisphosphate (Nourbakhsh et al.).

## The lipid signaling machinery is active under control conditions but can be modified by the action of hormones or elicitors via their inhibition of this basal pathway

It is now well-accepted that such control states or steady states correspond to a state were some signaling events are active and participate in the maintenance of a metabolic equilibrium that we consider as the quiescent state. It is possible to inhibit such activities. Ruelland et al., show that in Arabidopsis suspension cells salicylic acid (SA) treatment leads to an increase in phosphoinositides and to a significant 20% decrease in PA, indicative of a decrease in the products of the phosphoinositide dependent phospholipase C (PI-PLC). Interestingly they could identify genes for which the response to SA was dependent on phosphoinositides. In addition, they were able to identify genes whose response to SA could be mimicked by inhibitors of the PI-PLC pathway. They propose a model in which SA inhibits PI-PLC activity and alters levels of PI-PLC products and substrates, thereby regulating gene expression divergently (Ruelland et al.). Since then the authors also showed that abscisic acid (ABA) also participates in a decrease of the PI-PLC pathway activity, and this could account for the important ABA- and SA-transcriptome response overlap observed in Arabidopsis suspension cells (Kalachova et al., 2016). Likewise, xenobiotics or metals could exert their negative effects on plants by acting on the basal lipid signaling machinery. Pejchar et al., illustrate this in their work with non-specific phospholipases C (NPCs) showing that phospholipase C acts on structural lipids such as phosphatidylcholine. Aluminum exposure leads to decreased expression of *NPC4* and decreased NPC activity in Arabidopsis. The *in vitro* activity and localization of NPC4 were not affected by Al, thus excluding direct inhibition by Al ions or possible translocation of NPC4 as mechanisms involved in the NPC-inhibiting effect (Pejchar et al.). Interestingly, overexpressing NPC4 partly restored growth of Tobacco pollen tubes under Al stress. These observations suggest that NPCs play a role in the responses to Al stress because NPCs are likely inhibited by Al, and this inhibition is part of the deleterious effect of Al.

## Identification of putative new signaling molecules generated by these pathways

Inositol phosphates (InsPs) are linked to lipid signaling, as at least one portion of the inositol phosphate signaling pool is derived from hydrolysis of phosphatidyl inositol (4,5) bisphosphate, a substrate of some phospholipases C. The inositol pyrophosphates are a novel group of InsP molecules containing diphosphate or triphosphate chains (i.e., PPx) attached to the inositol ring. They are emerging as critical players in the integration of cellular metabolism and stress signaling in non-plant eukaryotes. Williams et al., review data suggesting a signaling role for these molecules in plants.

## Cross talk between lipid signaling pathways

The first step of sphingolipid synthesis, which uses a fatty acid and a serine as substrates, is critical for sphingolipid homeostasis. Fatty acids are released by the action of phospholipases A. Interestingly, manipulating the level of phospholipases A can impact the level of sphingolipids. Indeed, 3-keto-sphinganine, the product of the first step of sphingolipid synthesis, had a 26% decrease in leaves of mutants plants defective in expression of.*pPLAIII*β, a patatin-related phospholipase A, while a 52% increase could be measured in plants overexpressing it (Li et al.).

## Identification of the mode of actions of the signaling lipids

The lipids produced by the signaling pathways will trigger upstream signaling events. They can do so by binding to proteins, and thus modifying their localization and/or activity. But these lipids can also have effects on the physical properties of membranes. The work on diacylglycerol pyrophosphate (DGPP) and/or phosphatidic acid (PA) monolayers by Villasuso et al. illustrates this point. However, more work is necessary to fully describe the impact of signaling lipids on the physical states of membranes, such as in their fluidity, curvature, interaction with ions, and the consequent impacts on biological processes.

While most of the studies discussed so far concerned higher plants, we should not forget that lipid signaling pathways also exist in algae, including microalgae. Mikami provides a descriptive method to assess enzyme domain structures that provides suggestions as to the origin and evolution of signaling networks that regulate development and stress responses in terrestrial plants). Due to the importance of algae and microalgae in ocean ecosystems, and as potential industrial source of renewable biodiesel, the article by Mikami is an invitation to develop our research field with these fascinating models.

## Author contributions

All authors listed, have made substantial, direct and intellectual contribution to the work, and approved it for publication.

### Conflict of interest statement

The authors declare that the research was conducted in the absence of any commercial or financial relationships that could be construed as a potential conflict of interest.
